# Fall Risk Factors in Community-Dwelling Elderly Depending on Their Physical Function, Cognitive Status and Symptoms of Depression

**DOI:** 10.3390/ijerph120403406

**Published:** 2015-03-24

**Authors:** Magdalena Sylwia Kamińska, Jacek Brodowski, Beata Karakiewicz

**Affiliations:** 1 Department of Primary Health Care, Faculty of Health Sciences, Pomeranian Medical University in Szczecin, 48 Żołnierska St., 71-210 Szczecin, Poland; E-Mail: jabrod@wp.pl; 2 Public Health Department, Faculty of Health Sciences, Pomeranian Medical University in Szczecin, 48 Żołnierska St., 71-210 Szczecin, Poland; E-Mail: karabea@pum.edu.pl

**Keywords:** elderly falls, functional state, cognitive function, depression

## Abstract

Falls are the leading cause of unintentional injuries and injury-related disability, morbidity and mortality in the geriatric population. Therefore, they may also lower quality of life. The aim of this study was to analyze the fall risk factors in the community-dwelling elderly depending on their physical function, cognitive status and symptoms of depression. The study involved 304 individuals aged 65–100 years with a mean age of 78.6 ± 7.4. This survey-based study was conducted using the Geriatric Environmental Inquiry, the Barthel Scale (BS), the Abbreviated Mental Test Score (AMTS), the Geriatric Depression Scale (GDS) and the Tinetti Test (TT). There was a statistically significant correlation between the BS, the TT and the incidence of falls (*p* < 0.05). The number of falls correlated significantly with the results of the BS (R = −0.39), the GDS (R = 0.18), and the TT (R = −0.40). A statistically significant correlation was also noted between the TT results and the results of the BS (R = 0.77), the AMTS (R = 0.40) and the GDS (R = −0.37). The incidence of falls may significantly increase in people with a lower functional status, which may be related to cognitive process disturbances and lower affective functioning. A comprehensive geriatric assessment, related to all aspects of advanced-age patients’ efficiency, is recommended. Fall prevention strategies should include actions undertaken to evaluate and treat depression and cognitive disturbances.

## 1. Introduction

According to the definition accepted for the purpose of epidemiological research, a fall is an event which results in a person coming to rest inadvertently on the ground or floor or other lower level [1]. 

Epidemiological statistics indicate that every year falls happen to about 30% of adults aged over 65 and living in their own homes. However, about 50% of them don’t talk about their falls either to their caregivers or medical staff. What is more, elderly people staying in residential homes experience falls more often than those living in their own homes. Every year, falls happen to about 45% of people provided with long-term care, out of whom 40% experience multiple falls [1,2,3].

Among older adults, falls are the leading cause of both fatal and nonfatal injuries. The consequences of fall-related injuries among over 65-year-olds include: long-lasting disability, loss of autonomy, lower quality of life, and problems with organizing professional and non-professional care for this group of patients. Falls are the main cause of injury-related disability, morbidity and mortality in geriatric people [1,2,3,4,5,6].

In member countries of the European Union, the problem of injuries affects about 105,000 people. Furthermore, nearly 40,000 elderly people are pronounced dead due to falls. The rate of fall-related fatal injuries among those aged 60 or older is 36.8 per 100,000 in the United States, whereas in Canada it is 9.4 per 10,000 in the same age group [1].

In 2013, over 2,000,000 non-fatal injuries were treated in emergency departments, and more than 700,000 of them required hospitalization [4]. The hospital admission rate for injuries caused by falls among people over 60 and older in Australia, Canada, the United Kingdom of Great Britain and Northern Ireland is 1.6–3.0 per 10,000 citizens. In Western Australia and Great Britain, it is as high as 5.5–8.9 per 10,000 people. The most common fall-related causes of admission to hospital are hip joint fractures, traumatic brain injuries, and injuries to the upper extremities. It is also worth emphasizing that, in the case of the elderly, the period of hospitalization due to falls is considerably longer than hospitalization for other reasons [1].

Moreover, fall-related injuries among over 65-year-olds generate high costs for the health care and welfare sectors. In 2012, the direct medical costs of injuries caused by falls among people over 65 years of age, adjusted for inflation, was 30 billion dollars [1,2,3,4,5,6,7].

The incidence of falls rises with age and the worsening of general fitness. Regardless of the reasons, falls in advanced age have serious physical, mental, and socio-economic consequences. Falls usually result from the interaction and interference of factors categorized in four domains: biological, behavioral, environmental, and socio-economic [1]. 

Biological fall risk factors include fixed factors, such as age, gender, and race, and are closely related to involution changes and morbidity, which impair the functioning of the “postural control system” defined as internal causes of falls. External causes include behavioral, environmental, and socioeconomic factors. Behavioral fall risk factors are potentially modifiable, and include such issues as alcohol abuse, using inappropriate shoes, as well as physical inactivity. Behavioral factors also include polypharmacotherapy, and not taking into account the differences in drug pharmacokinetics and pharmacodynamics in the organism of an old person. Environmental factors refer to the development of private and public spaces (uneven terrain, architectural obstacles, and problems with transport). Socio-economic issues are: low income, low standard of living, limited access to health and social services, and the lack of social support [1].

Identification of the main fall risk factors may contribute to the implementation of preventive actions reducing the incidence of falls. These actions should form a part of an interdisciplinary process aiming at compensation of deficits in all spheres of human functioning. The specificity of geriatrics necessitates the provision of an overall solution to the problems of advanced-age patients. Therefore, the therapeutic and nursing management in the case of the elderly should be based on a comprehensive geriatric assessment (CGA). The literature describes a number of functional tests used to assess the risk of falls in people of advanced age. Their availability, the short time required for their execution, and the possibility of using them in any conditions, mean that they can be also applied on an outpatient basis [8]. The aim of this study was to analyze the fall risk factors in the community-dwelling elderly depending on their physical function, cognitive status and symptoms of depression.

## 2. Materials and Methods

### 2.1. Ethics Statement

All subjects gave their informed consent for inclusion before they participated in the study. The study was conducted in accordance with the Declaration of Helsinki, and the protocol was approved by the Ethics Committee of the Pomeranian Medical University in Szczecin, Poland (KB-0080/141/09).

### 2.2. Sample Size

The study involved 304 people aged 65–100 years, using outpatient health services provided by The Regional Centre of Occupational Medicine - West Pomeranian Prevention and Therapy Centre in Szczecin, North West Poland. Women constituted 77.3% of those analyzed, men −22.7%. The mean age was 78.6 ± 7.4.

### 2.3. Participants and Recruitment

Principal criteria for the selection of participants were: the availability of the patients at the moment of carrying out the study, no symptoms of the exacerbation of the co-existing diseases or other health problems, the mental condition of the patients making them able to cooperate during the study and understand the orders of the researcher, the ability of the surveyed to move without assistance, as well as conscious and voluntary consent to participate in the research. All subjects who met the above mentioned criteria were included in the study. All patients were acquainted with the nature, goals, and course of the study, and informed that their participation in the study was voluntary and they were free to resign at any stage. The examination time per patient was approximately 90–120 min and was adjusted to the individual needs of the patient. During the study the researchers applied the principles of effective interpersonal communication.

### 2.4. Data Collection

The study was conducted in conditions which guaranteed the accuracy of the measurement and elimination of interfering factors. The specificity of the research situation, as well as the characteristics of the participants and their health status were taken into account. The place where the participants were examined was suitable for people of advanced age. The study was conducted with respect for the patients’ dignity, intimacy, comfort and feelings of safety. 

The study was based on a diagnostics survey, conducted using the technique of an oral, open, individual categorized interview. The interview was conducted directly with the patients or indirectly with their professional or non-professional caregivers. Instruments employed in the study included the original Geriatric Environmental Inquiry. The questionnaire concerned the general assessment of socio-environmental and economic situation, as well as the health evaluation and circumstances of falls in the past. Additionally, observations were conducted using a non-standard, occasional, direct and spontaneous technique as a supplementary method. Another instrument used in the study was the Barthel Scale (BS) for measuring the ability to perform activities of daily living. This test enables the identification of three groups of patients depending on their self-reliance level. Individuals with scores of 0–20, 21–85, 86–100 points were classified as “very dependent”, “moderately dependent”, and “independent”, respectively. The Abbreviated Mental Test Score (AMTS) was applied to assess cognitive functions. This test enables the identification of three groups of patients: a group with seriously disturbed cognitive function (0–3 points), a group with moderate disability (4–6 points),and a group with normal mental efficiency (>6 points). The short version of the Geriatric Depression Scale (GDS) was used to assess the severity of depressive symptoms. Individuals with scores of 11–15 points are regarded as having severe depressive disorders, patients with scores of 6–10—moderate depression, and those with the score of 0–5 - no depressive symptoms. The Tinetti Test (TT) was employed to evaluate patients’ ability to walk and maintain balance. The TT enables the division of patients into three groups depending on the level of their dependence and the risk of falls. The group at the highest risk obtains the lowest scores (≤18). The group at moderate risk consists of people with scores of 19–23 points, which reflects moderate dependence and fall risk. The group at minimal risk is the one with scores of ≥24 points.

The last stage of the study involved the analysis of the documentation. The authors analyzed medical documents filled in by those taking care of a given patient (family doctor, family nurse, doctors of different specialties, physiotherapists) in order to verify information about health and nursing problems obtained during medical interview. The study was performed using a standard technique. The research instruments of choice were: the history of disease, the documentation of the nursing process, the results of consultation and diagnostic tests and hospital treatment information charts. 

### 2.5. Data Analysis

The research material was verified in terms of its completeness, reliability, and the correctness of its collection. Then, a statistical grouping was conducted in order to organize and systematize the material according to criteria determined by research problems and the aims of the study. The next stage was the codification of the research material, due to the complexity of the planned statistical procedures. The empirical material was processed using the calculation sheet MS EXCEL 2007. Statistical calculations were performed with the software “Statistica 10.0” (StatSoft Poland Inc., Krakow, Poland). The significance of differences between qualitative variables was assessed using the *t*-student test, and the Kolmogorov-Smirnov non-parametric test. The significance of differences between quantitative variables was assessed using the Spearman’s rank correlation coefficient or the Pearson correlation coefficient. The accepted level of statistical significance was α = 0.05. The authors also conducted the multifactor analysis of falls (>1), using the logistic regression model (Quasi-Newton estimation). The accepted level of statistical significance was α = 0.05.

### 2.6. Limitation of the Study

The limitation of this study was a relatively small study group, as well as the fact that this sample was not representative of the population of the center where the study was performed.

## 3. Results

Some 233 respondents had experienced at least one fall, out of whom 137 had experienced two falls, and 75—at least three falls. The highest number of falls per person in the year preceding the study was 12. It should be emphasized, however, that a high number of falls had not been recorded in medical documentation because the falls were not taken seriously by the patients and were not reported to medical staff. Furthermore, the health care center involved in this study has no procedure for documenting falls and does not keep a record of falls. Patients with the history of falls obtained significantly lower mean scores on the BS test and the TT test (Table 1). These patients were at the considerably higher risk of falls than those who had managed to avoid such events (Table 1).

**Table 1:** Barthel Scale (BS) and Tinetti Test (TT) results depending on fall incidence (Kolmogorov-Smirnov test).

Scale	Mean (SD)	Mean (SD)	Kolmogorov–Smirnov test		*p*
	Fallers n = 233	Non-Fallers n = 71	Maximum Negative Difference	Maximum Positive Difference	
Barthel Scale (BS)	85.49 (16.47)	91.40 (11.74)	0.00	0.24	0.00
Tinetti Test (TT)	19.18 (7.81)	22.67 (6.38)	0.00	0.24	0.00

There were no significant differences in the mean scores on the AMTS test and the GDS test between patients with and those without the history of falls (Table 2).

**Table 2:** Abbreviated Mental Test Score (AMTS) and Geriatric Depression Scale (GDS) results depending on fall incidence (*t*-student test).

Scale	Mean (SD)	Mean (SD)	*t*-Student Test	*p*
	Fallers n = 233	Non-Fallers n = 71		
Abbreviated Mental Test Score (AMTS)	8.39 (1.71)	8.32 (1.92)	0.31	0.75
Geriatric Depression Scale (GDS)	5.70 (3.32)	5.35 (3.67)	0.76	0.45

It was found that the patients with a previous fall were at a considerably higher risk of falls than those who managed to avoid such events (Table 1). Table 3, Table 4, Table 5, Table 6 and Table 7 show the correlations between the number of falls and the results of the Barthel Scale (BS), the Abbreviated Mental Test Score (AMTS), the Geriatric Depression Scale (GDS) and the Tinetti Test (TT). Three and more falls were classified to the same group (≥3).

**Table 3:** The correlation between the number of falls and the results of the BS (Pearson’s correlation coefficient).

Number of Falls	0		1		2		≥3		Total		Pearson’s χ^2^ Test	*p*
**BS**	**n**	**%**	**n**	**%**	**n**	**%**	**n**	**%**	**n**	**%**	39.16	0.00
0–20 points	0	0	0	0	1	1.61	0	0	1	0.32		
21–85 points	17	23.94	19	19.79	25	40.32	45	60	106	34.86		
86–100 points	54	76.06	77	80.21	36	58.06	30	40	197	64.82		
Total	71	23.36	96	31.58	62	20.39	75	24.67	304	100		

**Table 4:** The correlation between the number of falls and the results of the AMTS (Pearson’s correlation coefficient).

Number of Falls	0		1		2		≥3		Total		Pearson’s χ^2^ Test	*p*
**AMTS**	**n**	**%**	**n**	**%**	**n**	**%**	**n**	**%**	**n**	**%**	2.48	0.87
0–3 points	1	1.41	1	1.04	2	3.23	2	2.67	6	1.97		
4–6 points	10	14.08	12	12.5	5	8.06	10	13.33	37	12.17		
>6 points	60	84.51	83	86.46	55	88.71	63	84	261	85.85		
Total	71	23.36	96	31.58	62	20.39	75	24.67	304	100		

**Table 5:** The correlation between the number of falls and the results of the GDS (Pearson’s correlation coefficient).

Number of Falls	0		1		2		≥3		Total		Pearson’s χ^2^ Test	*p*
**GDS**	**n**	**%**	**n**	**%**	**n**	**%**	**n**	**%**	**n**	**%**	14.74	0.02
0–5 points	41	57.75	56	58.33	35	56.45	26	34.67	158	51.97		
6–10 points	21	29.58	34	35.42	23	37.1	38	50.67	116	38.16		
11–15 points	9	12.68	6	6.25	4	6.45	11	14.67	30	9.87		
Total	71	23.36	96	31.58	62	20.39	75	24.67	304	100		

**Table 6:** The correlation between the number of falls and the results of the TT (Pearson’s correlation coefficient).

Number of Falls	0		1		2		≥3		Total		Pearson’s χ^2^ Test	*p*
**TT**	**n**	**%**	**n**	**%**	**n**	**%**	**n**	**%**	**n**	**%**	50.11	0.00
0–18 points	15	21.13	24	25.0	30	48.39	52	69.33	121	39.8		
19–23 points	13	18.31	16	16.67	7	11.29	9	12.0	45	14.8		
24–28 points	43	60.56	56	58.33	25	40.32	14	18.67	138	45.4		
Total	71	23.36	96	31.58	62	20.39	75	24.67	304	100		

It was proved that there is a correlation between the number of falls and functional status (BS), cognitive status (AMTS), and affective functioning (GDS), as well as the risk of falls measured using the Tinetti Test (TT). Based on the obtained results, only the significance of the correlation between the number of falls and cognitive status (AMTS) was not confirmed. In other cases, the number of falls significantly correlated with scores obtained by the patients. The higher functional status correlated with a lower number of falls. Lower affective functioning was accompanied by a higher number of falls. Patients at the lower risk of falling occurred less frequently (Table 7).

**Table 7:** The correlation between the number of falls and the results of the Barthel Scale (BS), Abbreviated Mental Test Score (AMTS), Geriatric Depression Scale (GDS) and Tinetti Test (TT) (Spearman’s rank correlation coefficient).

Scale	Rs	*p*
Barthel Scale (BS) & number of falls	−0.39	0.00
Abbreviated Mental Test Score (AMTS) & number of falls	−0.06	0.26
Geriatric Depression Scale (GDS) & number of falls	0.18	0.00
Tinetti Test (TT) & number of falls	−0.40	0.00

The TT outcomes were compared with those of the BS, the AMTS and the GDS. A lower risk of falls was associated with higher functional and cognitive status, and less severe depression. There was a statistically significant correlation between the TT results and the results of the BS, the AMTS and the GDS. The BS results correlated most strongly with the fall risk assessment (TT) (Table 8). 

**Table 8:** The correlations between the TT and the BS, the AMTS and the GDS (Pearson’s correlation coefficient).

Scale	R	*p*
Barthel Scale (BS) & Tinetti Test (TT)	0.77	0.00
Abbreviated Mental Test Score (AMTS) & Tinetti Test (TT)	0.40	0.00
Geriatric Depression Scale (GDS) & Tinetti Test (TT)	−0.37	0.00

The authors used the multifactor logistic regression model, which enabled them to identify factors having an influence on the number of falls in the study group. They introduced values reflecting the number of falls, with “≤1” denoting 0 falls, and “1” denoting >1 falls (single falls had been incidental ones and had not caused any injuries). The multifactor logistic regression analysis showed that high scores on the BS and the TT corresponded with a lower number of falls, whereas high scores on the AMTS increased the probability of a higher number of falls in the study group. The GDS scores were not related to the occurrence of multiple falls (Table 9).

**Table 9:** Multifactor analysis-factors contributing to the occurrence of falls.

Scale	BS	AMTS	GDS	TT
**Score**	−0.04	0.17	0.03	−0.07
***p***	**0.02**	**0.04**	0.45	**0.00**
**OR**	0.97	1.19	1.03	0.93
**−95% CI**	0.94	1.01	0.95	0.88
**+95% CI**	0.99	1.39	1.12	0.98

BS—Barthel Scale, AMTS—Abbreviated Mental Test Score, GDS—Geriatric Depression Scale, TT—Tinetti Test; N = 304 (NF ≤ 1 = 0, n = 167; F > 1 = 1, n = 137); χ^2^ = 51.54, *p* = 0.00.

## 4. Discussion

The study presented here, demonstrated a significant relationship between functional status and a history of falls. The results reported by other researchers suggest that people who had experienced falls demonstrate a lower ability to perform everyday activities significantly more often [9,10]. It has also been confirmed that falls happen more often to people with lower functional status [11]. Some authors, on the other hand, claim that both poor physical fitness condition and a high degree of physical activity increase the risk of falls [12]. Furthermore, the studies of Kempen indicate that sight loss is a factor having profound effects on the performance of everyday activities, as well as the occurrence of depressive symptoms and feelings of anxiety [13].

The results described in this article show that the number of falls is related to functional status, and Harnbrook underlines the connection between the number of falls and motor disability [14]. According to some researchers, general physical status has an influence on the incidence of multiple falls [15]. There is also evidence that the history of falls, vision impairment, urinary incontinence, and functional limitations are important risk factors of multiple falls [2]. As Tinetti states, most falls happen during activities associated with a slight transfer of the center of gravity [16]. It has been scientifically substantiated that the incidence of falls and their effects on general physical condition depend on gender [17]. Thus, previous falls contribute to the limitation of physical activity, motor disability and a tendency towards further falls.

In this study, the risk of falls was significantly related to functional status. The above-mentioned issues have been confirmed by studies showing that a higher risk of falls is associated with a lower ability to maintain balance, which is typical of advanced age [18]. Disturbed gait and balance arecrucial predictors of falls [19]. It has been observed that people with an impaired ability to maintain the balance of their body have a higher tendency to falls [20].

Based on the obtained results, cognitive status and affective functioning do not have significant effects on the incidence of falls. A significant correlation between the number of falls and cognitive status was not confirmed either. Nonetheless, there are studies [21] which show a relationship between cognitive status and physical activity. Brito has proven that the occurrence of falls is accompanied by depressive symptoms and disturbances in balance [22].

The results presented demonstrate that cognitive status and affective functioning contribute to the risk of falls. The multifactor logistic regression analysis showed that high scores on the BS and the TT corresponded with a lower number of falls, whereas high scores on the AMTS increased the probability of a higher number of falls in the study group. The GDS scores were not related to the occurrence of multiple falls. This may be due to the fact that people with a higher functional and cognitive status are more likely to undertake physical activity. Some authors confirm that cognitive problems may lead to disturbed gait and balance, and thus increase the risk of falls [23,24]. Others emphasize the association between physical fitness, the risk of falls, and cognitive disturbances [25]. Vassallo claims that people with disturbances of primary cognitive processes have a disturbed walking pattern significantly more often [26]. Furthermore, according to the above mentioned authors, cognitive disorders are predictive of a higher incidence of falls and fall-related injuries in the future, and entail a higher mortality rate. Some studies show that people with cognitive disturbances and a history of falls, resulting in serious injuries, have lower motor efficiency [27]. Other studies prove that disturbed posture-balance, and especially such parameters as walking speed, standing balance and changes in the sitting-standing position, are related to a disturbed cognitive process [28]. Nevertheless, there are also reports suggesting that a program of exercises improving balance and the leg strength, in a group of patients with Parkinson’s disease, can contribute to a reduction in the incidence of falls, but only among patients at a mild stage of the disease [29]. Tinetti observed a significant correlation between the risk of falls and the incidence of depression [16]. However, other researchers suggest that a factor significantly increasing the risk of falls is not depression itself, but rather the administration of thymoanaleptiques [30]. Issues mentioned as important fall risk factors are also: a history of falls, visual disturbances, urinary incontinence, and using benzodiazepines [2]. 

## 5. Conclusions

The incidence of falls may significantly increase in people with lower functional status, which may be related to cognitive process disturbances and lower affective functioning. Since there was a relationship between the risk of falls and the lower functional status, cognitive status, and affective functioning in the analyzed group, a comprehensive geriatric assessment, related to all aspects of advanced-age patients’ efficiency, is recommended. Apart from improving the general health, fall prevention strategies should include actions undertaken to evaluate and treat depression and cognitive disturbances.
